# Experimental data reuploading with provable enhanced learning capabilities

**DOI:** 10.1126/sciadv.aeb1397

**Published:** 2026-04-10

**Authors:** Martin F. X. Mauser, Solène Four, Lena Marie Predl, Riccardo Albiero, Francesco Ceccarelli, Roberto Osellame, Philipp Petersen, Borivoje Dakić, Iris Agresti, Philip Walther

**Affiliations:** ^1^ University of Vienna, Faculty of Physics, Vienna Center for Quantum Science and Technology (VCQ), Boltzmanngasse 5, Vienna 1090, Austria.; ^2^ University of Vienna, Vienna Doctoral School in Physics (VDSP), Boltzmanngasse 5, Vienna 1090, Austria.; ^3^ Master Quantum Engineering, Ecole Normale Supérieure, Université PSL, 75005 Paris, France.; ^4^ Istituto di Fotonica e Nanotecnologie, Consiglio Nazionale delle Ricerche (IFN-CNR), Piazza L. Da Vinci 32, 20133 Milano, Italy.; ^5^ University of Vienna, Faculty of Mathematics and Research Network Data Science @ Uni Vienna, Kolingasse 14-16, Vienna 1090, Austria.; ^6^ Institute for Quantum Optics and Quantum Information Sciences (IQOQI), Austrian Academy of Sciences, Boltzmanngasse 3, Vienna 1090, Austria.; ^7^ QUBO Technology GmbH, Vienna 1090, Austria.

## Abstract

The past decades have seen the development of quantum machine learning, stemming from the intersection of quantum computing and machine learning. This field is particularly promising for the design of alternative quantum (or quantum inspired) computation paradigms that could require fewer resources with respect to standard ones, e.g., in terms of energy consumption. In this context, we present the implementation of a data reuploading scheme on a photonic integrated processor, achieving high accuracies in several image classification tasks. We thoroughly investigate the capabilities of this apparently simple model, which relies on the evolution of one-qubit states, by providing an analytical proof that our implementation is a universal classifier and an effective learner, capable of generalizing to new, unknown data. Hence, our results not only demonstrate data reuploading in a potentially resource-efficient optical implementation but also provide theoretical insight into this algorithm, its trainability, and generalizability properties. This lays the groundwork for developing more resource-efficient machine learning algorithms, leveraging our scheme as a subroutine.

## Teaser

Experiments show photonic data reuploading enables universal classification with learning and generalization guarantees.

## INTRODUCTION

The past decades have witnessed the emergence of quantum computing as a new paradigm to solve problems, with the promise of outperforming standard methods. In particular, the discovery of Shor’s and Grover’s algorithms (*1*, *2*) has ultimately proven that quantum computers can efficiently tackle computational problems that are hard for any known classical algorithm. However, tasks with a wide range of applications, like prime numbers factorization, are still out of reach for the current state of the art of quantum hardware, and a quantum advantage has been only demonstrated for problems with no practical use, like Boson Sampling and Random Circuit Sampling (*3*–*7*). On the other hand, a research field that has attracted a flurry of interest stems from the combination of quantum computing with machine learning (*8*, *9*). In this context, the aim has been to create a fruitful intersection, where quantum computers can provide a boost with applicative implications, by enhancing the performance of standard machine learning algorithms. Mutually, the latter can offer methods to gain deeper insights on quantum systems, either by postprocessing large amount of data or by directly dealing with quantum data without the need of classical description.

**Fig. 1.**
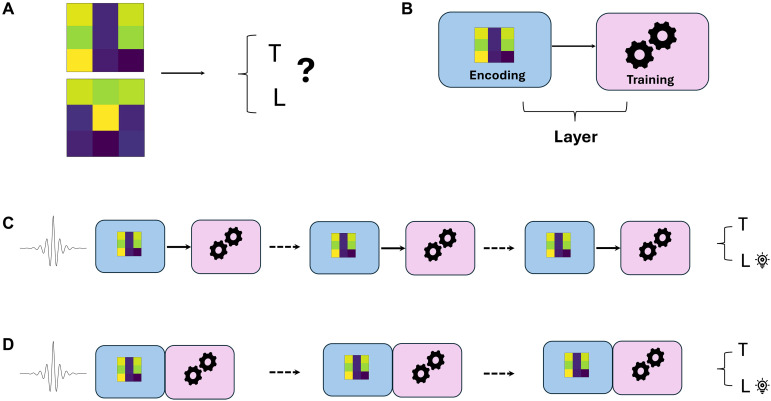
Image classification through data reuploading. (**A**) The classification tasks we tackle in this work amount to assign a given image to one, out of two, classes. Here, we report the example of tetromino images, picturing either an L or a T, and our model needs to understand which letter is represented. (**B**) The data reuploading algorithm is composed by the sequence of many layers. One layer is composed by the encoding of the input data (in this case, the image we want to classify) in a qubit state, followed by a random rotation, whose parameters are optimized through a training phase. (**C**) The initial qubit state, pictorially represented by a wavepacket, which is fixed, is injected into a sequence of many layers (alternating encoding and rotations). The optimal rotations are found after a training phase, ensuring that the output state holds the information of whether the input belongs to the first or second class. In the original proposal, the encoding and the rotations are implemented in two separate gates, and we refer to this scheme as original. (**D**) A variant of the algorithm merges the encoding and the rotation in the same gate. On one side, this reduces the length of the circuit, but, on the other, it hinders the generalizability of the method. We refer to this scheme as compressed.

Considering the challenges involved in the implementation and control of a high number of qubits and the possible experimental errors, it is crucial to identify the lowest amount of quantum resources, which allows us to tackle relevant tasks. The answer to this question was recently offered by the development of a quantum machine learning algorithm called data reuploading (*10*), whose concept is depicted in Fig. 1.

Despite its apparent simplicity, this model achieves highly complex mappings of the input data, constituting an alternative to kernel methods (*11*–*14*), and it has been proven to be a universal approximator (*10*, *15*). Furthermore, the reuploading of the input data circumvents the no cloning theorem (*16*), which prevents quantum models from easily copying and retrieving the input multiple times, as it would happen in the classical case. This model has been widely investigated from the theoretical point of view (*15*, *17*, *18*), and it was also implemented on several quantum platforms (*19*–*25*). However, most of the reported experiments slightly diverge from the theoretical proposal, since they express each encoding of the input data followed by a qubit rotation as a single gate (we will refer to this model as compressed). The two schemes, i.e., the original and the compressed one, are shown in Fig. 1 (C and D), respectively. Although this might seem a minor or even nonexisting difference, it has a huge impact on the complexity of the learning model, and it markedly affects its generalizability and trainability (*26*–*28*). To properly understand this concept, let us consider that the quality of a learner is defined by two aspects: its ability to remember labeled inputs and how well it can generalize its operation to new unknown data. Hence, choosing the best learning model will be a balancing act between an overly expressive and slightly underexpressive one, i.e., the complexity of the learner should match the complexity of the task. Moreover, we implemented the data reuploading model in a photonic platform using one-photon states distrbuted over two spatial modes. This setup uses a minimal set of experimental resources and provides a convenient parametrization of the optimization problem, granting provable advantages in particular on its trainability.

**Fig. 2.**
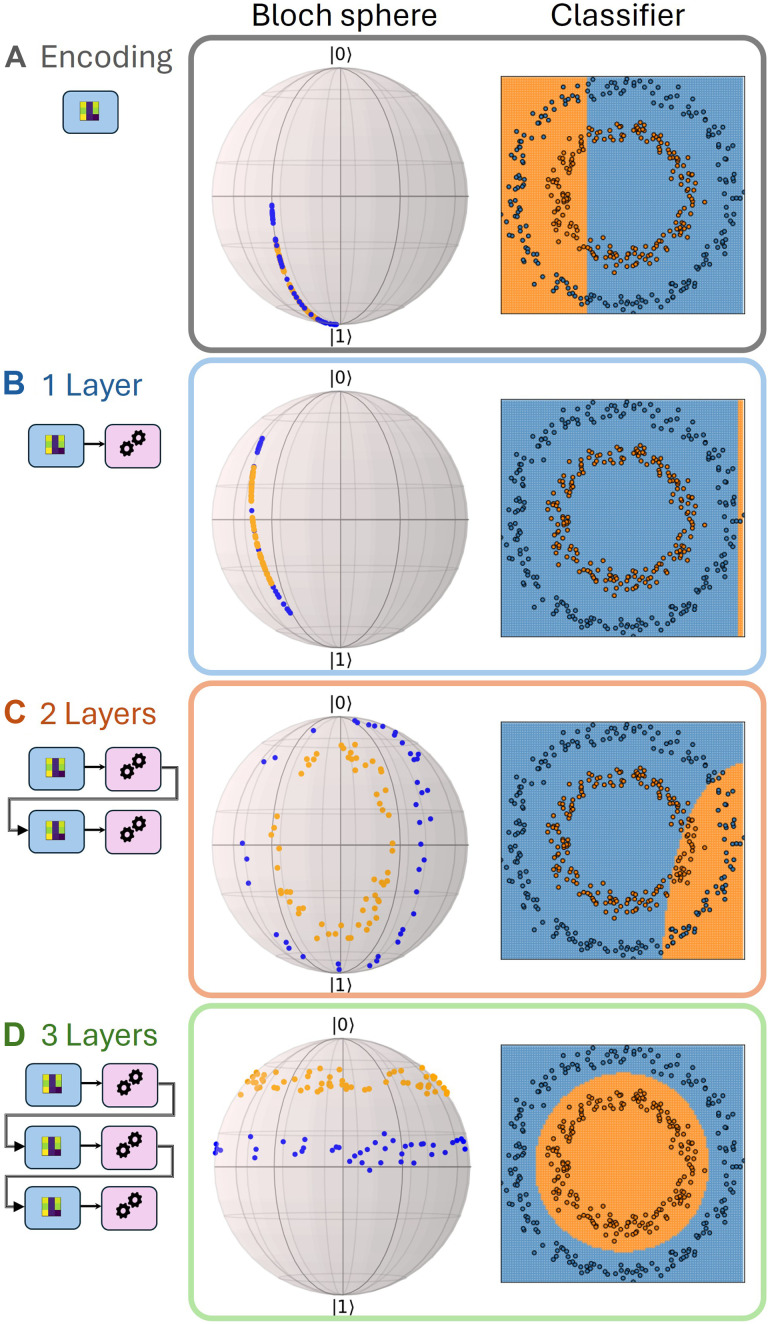
Photonic data reuploading. We show the working principle of our photonic data reuploading algorithm, illustrating the example of the circle dataset, where we want our model to distinguish between inner points and external ones. We start with the encoding of the data points (**A**), and we show the effect of this first gate on the initial state on the Bloch sphere. The blue (yellow) color represents the correct label of the point. On the right, we show the prediction of the algorithm if we would stop the algorithm here, gaining a poor performance. Again, the blue (yellow) color of the dot represents the true label, while the background shows the label predicted by the model. In other words, all the points in the yellow (blue) region are classified as yellow (blue). Then, we add the first rotation (**B**), and we show the action of the second (**C**) and third (**D**) layers. It is visible from the shape of the colored regions in the background how, increasing the number of layers, the model gets more complex and nonlinear. In this case, already after three layers, the algorithm successfully classifies the circle dataset.

In this work, we demonstrate a realization of a data reuploading scheme on a tunable integrated photonic processor (*29*) fabricated by femtosecond laser waveguide writing (*30*), which, because of its architecture, ensures that the algorithm is designed according to the initial theoretical proposal, i.e., separating the input encoding from the optimizable part. We show its working principle in Fig. 2. From a theoretical point of view, we go beyond the most recent theoretical reported work on data reuploading (*31*) and prove that our universal approximator succeeds in tasks in which previous implementations fall short while also showcasing better convergence to the optimal parameters, during the training phase. Furthermore, from the experimental point of view, we show its performance on a vast set of binary image classification tasks of increasing complexity. These results offer deep insights on the generalizability of quantum machine learning models and show how quantum architecture can offer inspiration to formulate optimization problems in a more convenient way. This work opens also the way to optical computing models, which promise to bring more energy-efficient algorithm executions (*32*).

## RESULTS

### Photonic data reuploading

The model that we design and implement in this work follows the original proposal of data reuploading (*10*). Its structure amounts to consecutive rotations $U\left(\theta_{1},\theta_{2},\theta_{3}\right)$ applied to a single qubit input, which we denote as $|\Psi_{\text{in}}\rangle$. Conceptually, in this sequence, we can distinguish two kinds of rotations: (i) encoding and (ii) processing. The first is required to upload the input data to be classified into the model; the second, instead, has the scope of implementing a nontrivial feature map of the input into an output state $|\Psi_{\text{out}}\rangle$, so that a linear classification is sufficient in the end to perform the desired task.

Hence, from a mathematical point of view, the overall operation of the model is the following

$$|\Psi_{\mathrm{out}}\rangle =U\left(\vec{x},\vec{\xi }\right)|\Psi_{\text{in}}\rangle =\prod_{l=1}^{L}\left[U_{l}\left(\vec{\theta }\right)U\left(\vec{x}\right)\right]|\Psi_{\text{in}}\rangle$$ (1)

where $\vec{x}$ represents the initial features of the classical input data, which will define all of the encoding unitaries, $\vec{\xi }=\left({\vec{\theta }}_{1},\ldots ,{\vec{\theta }}_{L}\right)$ is a vector containing the optimizable parameters, which will amount to the processing rotations. Then, a pair $U_{l}\left(\vec{\theta }\right)U\left(\vec{x}\right)$ composes a layer of the model (see Fig. 1B). Let us note that a single qubit rotation has three free variables, but in this work, we will consider the implementation through Mach-Zehnder interferometers (MZIs). These implement a restricted two-parameter subset of the Special Unitary Group of degree 2 [SU(2)] transformations. This implies that, in case the considered data points have a higher dimension than 2, the encoding gate will be given by a composition of encoding rotations, i.e., *d*/2 (or, if *d* is odd, [*d*/2] + 1). In our model design, we alternately use an encoding rotation and a trainable one. This implies that each layer will be composed by *d*/2 (or [*d*/2] + 1) encoding rotations and *d*/2 (or [*d*/2] + 1) trainable ones. Hence, for *L* layers, we will have a total depth, or number of gates/Mach Zehnders, of 2*L*. In the end, the output state is projected on the computational basis (∣0〉, ∣1〉), constituted by the eigenvectors of the Pauli *Z* operator. The result will be a bidimensional vector (p,1−p), where $p=\mathrm{Tr}\left(|0\rangle \langle 0|\Psi_{\text{out}}\rangle \langle \Psi_{\text{out}}|\right)$. These data are then classically postprocessed through linear discriminant analysis (LDA) (*33*), to find the linear separation between the two considered classes.

Hence, the classical algorithm will find a threshold τ, such that, if *p* ≥ τ, with

$$p=\mathrm{Tr}[|0\rangle \langle 0|U\left(\vec{x},\vec{\xi }\right)|\Psi_{\text{in}}\rangle \langle \Psi_{\text{in}}|U^{†}(\vec{x},\vec{\xi })]$$ (2)

the data point $\vec{x}$ will be assigned to class 1, otherwise to class 2.

Analogously to standard artificial neural networks, the higher the number of layers the model will display, the better its representation capabilities will be, implying an overall more powerful classifier (*15*), as it is also visible from Fig. 2. While this might constitute an issue for platforms like superconducting qubits, due to their limited coherence time, it is particularly suitable for photonic implementations.

Let us consider that, although in this model the input data points are introduced linearly in the rotations, the overall feature map will be anyway nonlinear, due to the structure of these gates, ensuring that it can potentially implement a universal classifier (*15*, *17*, *18*). To show such a nonlinearity, let us consider that in our model each layer of the classifier is implemented by a pair of MZIs, operated in the path degree of freedom (see Methods). Such a gate can implement any rotation of the Bloch sphere, and it features two external phases (ϕ_1_ and ϕ_2_) and an internal one (θ) and is represented through the following Jones matrix

$${\mathrm{ie}}^{i\frac{\theta }{2}}\left(\begin{array}{cc} e^{i\left(\phi_{1}+\phi_{2}\right)}\text{sin}\left(\frac{\theta }{2}\right) & e^{i\left(\phi_{1}\right)}\text{cos}\left(\frac{\theta }{2}\right)\\ e^{i\left(\phi_{2}\right)}\text{cos}\left(\frac{\theta }{2}\right) & -\text{sin}\left(\frac{\theta }{2}\right) \end{array}\right)\left(\begin{array}{c} 0\\ 1 \end{array}\right)$$ (3)

where we inject the input state ∣1〉, which, in the dual-rail encoding, corresponds to the presence of a photon on one of the spatial modes of the MZI (see Methods). From Eq. 3, it is visible that a projective measurement on the *Z* basis will produce probabilities that depend nonlinearly on the phases. Let us note that, for deriving Eq. 3, we considered the dielectric beam splitter.

However, since our input is not a superposition of ∣0〉 and ∣1〉, we need one less degree of freedom and we can consider ϕ_2_ = 0. So, for the sake of notation, from now on, we will omit the subscripts and indicate ϕ_1_ simply as ϕ. For the same reason, let us note that we are not sensitive to the ϕ_1_ of the first Mach-Zehnder of our circuit, as it can be seen from Eq. 3.

Then, to encode our data, assuming that it is constituted by bidimensional points $x=\left\{x_{1},x_{2}\right\}$, we use the following map: $\phi =x_{1}\frac{\pi }{2}$ and $\theta =x_{2}\frac{\pi }{2}$ (see the section “Experimental apparatus”). In the case of higher dimensional input data, e.g., $x=\left\{x_{1},\ldots ,x_{n}\right\}$, if *n* is an even number, we proceed by encoding the coordinates in consecutive unitaries, in the following way

$$\Pi_{j=0}^{\left\lceil \frac{n}{2}\right\rceil -1}U\left(\theta_{2j},\theta_{2j+1}\right)U\left(x_{2j},x_{2j+1}\right)$$ (4)

Instead, if *n* is odd, an extra layer needs to be added, implementing $U\left(\theta_{n},\theta_{n+1}\right)U\left(x_{n},0\right)$. Hence, the lowest number of gates required to encode classical data of dimension *n* amounts to $\frac{n}{2}$, and the size of the array of trainable parameters is at least as big as one of the input. We will refer to the building block of our model, reported in Eq. 4, as base layer block (*34*).

### Trainability, generalizability, and universality of the model

Similar to other supervised machine learning models, the performance of data reuploading amounts to the accuracy with which the algorithm can reproduce the labeling of training data and of test data, which is unknown. Although both are important, the latter is more relevant, since the main purpose of machine learning models is to deal with unknown inputs, namely, they should have good generalization properties.

Several tools have been introduced to quantify generalization (*26*–*28*), but one of the most important is the Vapnik-Chervonenkis (VC) dimension (*35*–*37*). This parameter is tightly related to the complexity and expressivity of the model itself.

More formally, the VC dimension of a class of functions H (in our case those that our classifier can implement) is defined as the size of the largest set of points for which, for every possible assignment of labels (0 or 1), there exists a function in H that classifies them correctly. If we now consider the theoretical framework of probably approximately correct (PAC) learning (*38*), then the minimum amount of data required to achieve a given error rate is proportional to the VC dimension. This implies that, for a model featuring an infinite VC dimension, no finite amount of data suffices to correctly classify unknown data. Moreover, another side effect of high complexity is that the training procedure could be extremely inefficient or even impossible due to a very irregular optimization landscape for the loss function (*39*). Hence, for any task, a trade-off must be found between the expressivity of the model and its generalizability.

To mathematically show the implications of the different VC dimensions in the two schemes, let us consider the functions that a single layer can implement in each of the two models. In the compressed case, the functions that can be implemented have the following form (see note S1 for derivation)

**Fig. 3.**
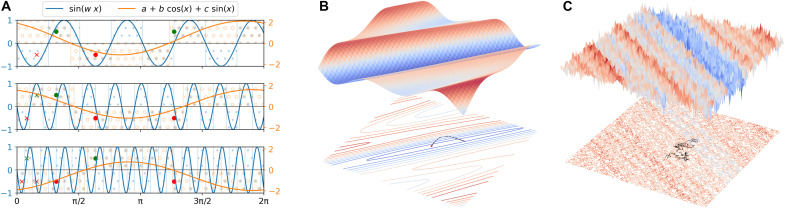
Generalizability and trainability of data reuploading models. (**A**) Visual representation of how the compressed data reuploading scheme (indicated with the blue curve) can correctly label arbitrarily many training data points, while the original one (studied in this work) can only do the same for up to 3 points. This implies that the compressed scheme has an infinite VC dimension, while ours is finite, i.e., VCdim = 3 (for one layer). This ensures that our model can generalize to test data points. The marked points indicate the training dataset, while the color and vertical position indicate the true label. The shaded areas indicate the classification result for the two learning models. The shape of the marked points indicate if both models where able to classify all points at that horizontal position correctly (dot) or only the blue sinusoidal classifier with tunable frequency (cross). (**B** and **C**) Loss landscape in the two cases: original (B) and compressed (C). Two layers are considered for both images, and the loss function has been projected to two dimensions via a principal components analysis (PCA) for visualization purposes. This demonstrates how the trainability of the model is influenced by its VC dimension. In addition, one possible optimization path using the Adam optimizer is shown on the lower heatmap of the two loss landscapes.

H={x↦sin(ωx)∣ω∈ℝ} (5)

and it can be shown that H has an infinite VC dimension. This is pictorially represented in Fig. 3, where we consider a toy model, where the data points are given as *X_i_* = 2*^i^*, and *i* represents an index ranging from 0 to *N*. The corresponding labels are drawn randomly from $y_{i}\in \left[0,1\right]$. It is visible that, with the model of Eq. 5, it is possible to reproduce any labeling choosing the following frequency ω

$$\omega =2\pi \sum_{i=0}^{N}\frac{y_{i}}{2^{i}}$$ (6)

This proves that the VC dimension of this learning model is infinite, as it was also demonstrated in (*40*).

On the contrary, the original scheme, when featuring a single layer, can only reproduce the following set of functions

H={x↦sign[a+bcos(x)+csin(x)]∣a,b,c∈ℝ} (7)

which can be described by the following periodic interval classifier

$$H=\left\{x\mapsto 1_{S=\left[a+2\pi n,b+2\pi n\right]}|a,b\in \mathbb{R};\forall n\in \mathbb{Z}\right\}$$ (8)

This implies that all the points in the interval [a+2πn,b+2πn] are classified as belonging to class 1 and those outside to class 2. Both Eqs. 7 and 8 display VCdim(H)=3 (see note S1). Hence, as it is shown in Fig. 3, the model can correctly reproduce arbitrary labelings of only 3 points. Then, when considering two layers, the interval S becomes [a+2πn,b+2πn]∪[c+πn,d+πn] with a,b,c,d∈ℝ and ∀n∈ℤ, with a corresponding VC dimension VCdim(H)=5. In general, it holds that 𝓁 layers lead to a VC dimension of 2𝓁+1. Concretely, we can choose 2𝓁+1 distinct points $x_{1},\ldots ,x_{2𝓁+1}$ in an interval [*a*, *b*] and show that they can be completely separated according to their label (shattered) by 𝓁 periodic interval classifiers. To show this, let us consider a set of labels $y_{1},\ldots ,y_{𝓁+1}\in \left\{0,1\right\}$. All points can be mapped to the label 1 by an element of the hypothesis class. Further, we can assume without loss of generality that *y_i_* = 0 for at least one element *i*, and by the periodicity of the classifiers, we can assume *i* = 1. Therefore, $\left\{x_{1},\ldots ,x_{2n+1}\right\}$ can be shattered by periodic interval classifiers on [*a*, *b*] if $\left\{x_{2},\cdots ,x_{2n+1}\right\}$ can be shattered by nonperiodic interval classifiers on $\left[\left(x_{1}+x_{2}\right)/2,b\right]$. We assume again, without loss of generality, that $x_{1}<x_{2}<\cdots$. This follows by (*40*), showing that the VC dimension of the periodic interval classifier is at least 2*n* + 1. The proof is completed by observing that for any $\left\{x_{1},\ldots ,x_{2n+2}\right\}$, the alternating label sequence {0,1,…,0,1} cannot be produced using *n* periodic interval classifiers.

The same analysis can be performed with higher-dimensional input data, also leading to a finite VC dimension, that is growing with the amount of layers (see note S1). Hence, from a theoretical point of view, the original scheme, implemented in our work, is proven to generalize to unknown data. Let us note, however, that the finiteness of the VC dimension does not imply a nonuniversality of the learning model. The analysis of VC dimension not only provides a formal guarantee of learnability but also has practical relevance for understanding the limitations of different architectures. While our proof is presented for a simplified one-dimensional setting, the conclusions extend to higher-dimensional encoding because the underlying structure of the algorithm remains unchanged. As established in (*40*), algorithms with infinite VC dimension lack generalization guarantees, making architectural design choices critical for practical success.

To gauge the effectiveness with which the algorithm can find the optimal parameters during training, let us consider the smoothness of the loss landscape. Comparing the two schemes, visually represented in Fig. 3, we can notice a marked difference in the appeal of the two loss landscapes indicating possible problems for both trainability and generalizability of the compressed one. Learning models with flatter minima, like the original scheme, tend to generalize better to new data and tend to be less sensitive to noise (*41*–*43*).

This is tightly related to the sharpness of the minima, namely, how quickly the loss function changes around them, which can be measured by its curvature (second derivative or Hessian). This can quantify the difficulty of landing in such a minimum, since it requires carefully balancing the learning rate: Too large may cause overshooting, while too small leads to slow progress. If we reconsider the worst-case dataset, which was used to demonstrate that the VC dimension of the compressed model is infinite, then we can numerically determine the sharpness of the minima in the two situations, in addition to visually interpreting the loss landscape. This is, as previously mentioned, related to the second derivative (Hessian) of the loss function. Note that since the model is not guaranteed to converge to the optimal minimum, the sharpness can only be evaluated in terms of an interval. In this context, its lower bound can be quantified by the largest eigenvalue of the Hessian matrix at the minimum itself (*44*). In our case, our numerical approximation gave ≈0.23 for the original scheme and ≈7.23 × 10^12^ for the compressed scheme. This thereby suggests markedly sharper minima for the compressed scheme.

To summarize, our model form, which separates the encoding and processing parts, is mathematically guaranteed to be an effective learner (according to the PAC learning definition). By this, we mean that the algorithm can, with high probability, output a hypothesis whose error is within a specific bound of the optimal, given a finite number of samples and reasonable computational resources. Hence, an arbitrary lower bound on the error rate on a test set can always be reached, with a finite number of samples. This is not guaranteed to happen in the compressed scheme. Let us anyway point out that both learning models are universal approximators (*20*, *31*) (see note S2), while differing markedly in their respective generalization and trainability properties.

### Performance of the model

We tested our photonic data reuploading scheme on four different tasks, of increasing complexity. These correspond to a particular dataset, whose elements we want to classify: (i) the circles, (ii) the moons, (iii) the tetrominos, and (iv) the Overhead Modified National Institute of Standards and Technology dataset (MNIST).

**Fig. 4.**
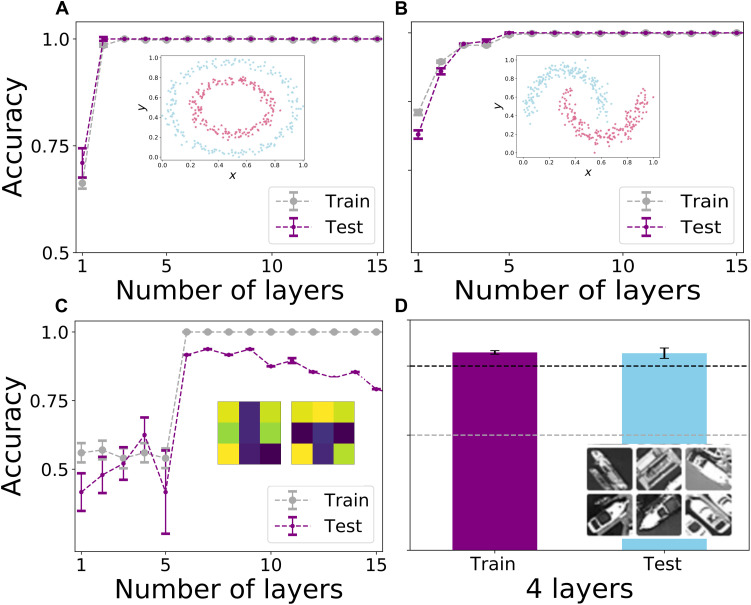
Experimental classification accuracies. We report the classification accuracies obtained through our photonic data reuploading model for the classification of four classes of images: (**A**) circles, (**B**) moons, (**C**) tetromino, and (**D**) images from the Overhead MNIST dataset, representing ships and cars. The gray dots represent the training accuracy, while the purple ones represent the testing. The first two sets are composed of bidimensional data [which are the (*x*,*y*) coordinates on the plane], so the base layer block is only composed of two MZIs. In this case, we used 400 samples as training and 100 as test. The tetromino images, instead, are composed of 9 pixels (containing a number between 0 and 1), implying that we need a base layer block of 10 MZIs. In this case, we used 100 noisy data samples for training and 48 noiseless for test. Regarding the Overhead MNIST images, instead, they were composed of 28 × 28 pixels, reduced to 20 parameters through PCA (*47*, *48*). Hence the base layer block amounts to 20 MZIs. In this case, we used 1776 data samples for training and 222 for test. For the cases (A) and (B), it can be seen that the accuracy in the prediction gets better when increasing the number of layers, reaching the maximum after three layers. For the tetromino dataset, we see a drop in the test accuracy, due to overfitting when the number of layers (or free parameters) is too high. In the case of the Overhead MNIST images, we report the accuracy obtained for four layers. The error bars are obtained through a Monte Carlo simulation, considering 1000 repetitions for each training and testing, where we consider that the underlying statistics for our photon detection is Poissonian, and represent one SD.

The circles and moons dataset feature bidimensional data. The former is created using the implementation of the Python library scikit-learn (*45*). Hereby the scaling factor between the inner and outer circles is 0.6 with a 0.05 Gaussian noise. The second dataset, instead, features two interleaving half circles. The points lying on the first curve are labeled as 1, and the ones lying on the second are labeled as 2. To generate this dataset, again, the implementation from scikit-learn (*45*) was used, with a 0.1 Gaussian noise. In both cases, the same setting, including the noise level, are used for both training and testing data. These data points, being bidimensional, can be encoded by a single MZI. Hence, in this case, the base layer block is constituted by two interferometers (one for encoding and one with trainable parameters). In Fig. 4 (A and B), we show the results for the training and test accuracy, and we can see how three layers are sufficient to achieve a perfect classification.

The tetromino dataset, instead, features 3 × 3 pixel figures, representing “T” and “L” letters, corresponding to classes 1 and 2, as in Fig. 4C. These letters are represented by black and white pixels. Then, to make the task more complex, we add uniform background noise bounded between −0.1 and 0.1 to these 3 by 3 matrices and we add to the dataset also the negative of the images. These data points contain nine binary values and hence require five MZIs to be encoded. Therefore, the base layer block will display 10 MZIs, i.e., five times the sequence of encoding and training. In Fig. 4C, we show the results for the training and test accuracy. In this case, we can see that the test accuracy is reduced, for a too high number of layers. An excessive complexity of the model leads to overfitting the dataset.

For the Overhead MNIST dataset (*46*), we considered the task of classifying between ships and cars. In this case, the images were preprocessed through principal components analysis (PCA) (*47*, *48*) featuring 20 parameters. Hence, in this case, the base layer block was composed by 20 interferometers. Given the very high amount of optimizable parameters, we performed the experiment only up to four layers, and the corresponding accuracy is shown in Fig. 4D.

In this last case, to separate the contribution of our model, from the one obtained by the PCA reduction, we tested the performance of the LDA classifier both feeding it directly with the uncompressed images, as well as with the PCA-reduced ones, without using our model. In the first case, the LDA achieves a good training accuracy (≈97.46%) but a poor one on the test set (≈74.32%). The preprocessing enhances the test accuracy to ≈81.53% while losing on the training one ≈82.27%. Hence, our model guarantees a higher accuracy on both training and testing sets. The role of the LDA itself in the classification pipeline does not account for the observed performance improvements but rather functions as a tunable threshold for the classification (see note S4).

The uncertainty was evaluated considering the effect of finite photon statistics, which follows the Poisson distribution. Hence, the error bars plotted in Fig. 4 show the SD obtained in the accuracy of the model, considering photon counting fluctuations. It can be seen that increasing the number of layers make our model more robust to this type of noise.

It is noteworthy that we consider only this type of noise as it is the dominant source of uncertainty in our experiment. Considering the integrated processor, thermal cross-talk and fabrication imperfections are accounted for during the calibration of the device, which ensures a fidelity of 0.9934 ± 0.0048 in the implementation of Haar unitaries (*29*). In addition, phase stability has been characterized in similar devices, with short-term fluctuations as low as 23.5 mrad over 13 hours of operation (*49*), with no long-term drift observed. Also regarding generation and detection, considering the fact that ours is a one-photon experiment, imperfections are negligible, with respect to shot noise.

## DISCUSSION

In this study, we have demonstrated the implementation of a data reuploading scheme on an integrated photonic platform, achieving high accuracies in several image classification tasks of increasing complexity. Our results provide both experimental and theoretical insights into the capabilities of data reuploading, particularly in terms of the implementation of that algorithm and the influence on trainability and generalizability.

Our experimental results show that with a minimum of resources, i.e., one qubit states and a sequence of MZIs, the data reuploading scheme can be effectively used for binary image classification tasks. The theoretical analysis complements these findings by proving that our implementation is not only a universal approximator but also an effective learner, offering a smoother optimization landscape, improved noise stability, and, most importantly, provable generalizability.

Compared to previous implementations, our approach maintains a clear separation between encoding and processing gates. This distinction is crucial as it enhances the trainability and generalizability of the learning model. As we show, compressed implementations of the data reuploading can bring issues like overfitting, instability, and a lack of provable generalization properties, due to their infinite VC dimension.

Despite the promising results, the above statements about trainability and generalizability for the compressed scheme are worst-case examples and might not be as marked in other tasks. These worst-case results are not merely theoretical but rather define the boundary conditions for learnability and motivate strategies such as regularization or inductive biases to effectively reduce complexity in compressed models. This connection between theoretical bounds and practical design highlights why complexity analysis is essential for guiding the development of scalable and reliable quantum learning models. Lower VC dimension is preferable when the amount of training data is limited. This indicates that architectural choices should restrict the number of reuploading layers to enhance robustness and mitigate overfitting.

A most relevant aspect of our model lies in the fact that it can be implemented both exploiting coherent light input and with single-photon states. The first is the most convenient when the model is implemented as a standalone, given the proven higher efficiency of optical-based computing (*32*, *50*), while the second can be used as a building block for more complex quantum computing architectures (*51*, *52*).

Future research should focus on investigating the exact effect if data reuploading is used as a subroutine. In addition, from the experimental side of this work, one should focus on scaling photonic data reuploading models to handle more complex and higher-dimensional data. This likely includes expanding to larger photonic systems and continue to investigate training on the photonic hardware (*53*, *54*), as it was already done for one qubit in this paper. Further, one could study regularization schemes to also have provable generalization capacities for the compressed scheme.

From an intuitive standpoint, the smoother loss landscape observed in our architecture should also contribute to improved training behavior under realistic noise conditions. This expectation is supported by our successful training runs directly on the chip. However, a rigorous comparison between models and detailed numerical simulations incorporating realistic noise sources remain beyond the scope of this work and represent an important direction for future research.

Regarding scalability, the complexity of the data reuploading model grows linearly with the number of layers, while the VC dimension scales as 2*l* + 1. However, in photonic hardware, photon propagation losses increase exponentially with the number of cascaded interferometers, making the implementation of very deep circuits highly impractical. For this reason, for high-dimensional dataset, dimensionality reduction techniques remain essential. Alternatively, coherent light inputs offer a practical solution for large-scale implementations, given their robustness to losses and compatibility with energy-efficient optical platforms (*32*).

To conclude, our study demonstrates the potential of data reuploading schemes in quantum machine learning, particularly when implemented on photonic platforms. The findings highlight the importance of maintaining a separation between encoding and processing gates to ensure model generalizability and trainability. Furthermore, the optical implementation lays the groundwork for future energy-efficient machine learning models, whether quantum inspired or fully quantum, for practical applications.

## METHODS

### Experimental apparatus

**Fig. 5.**
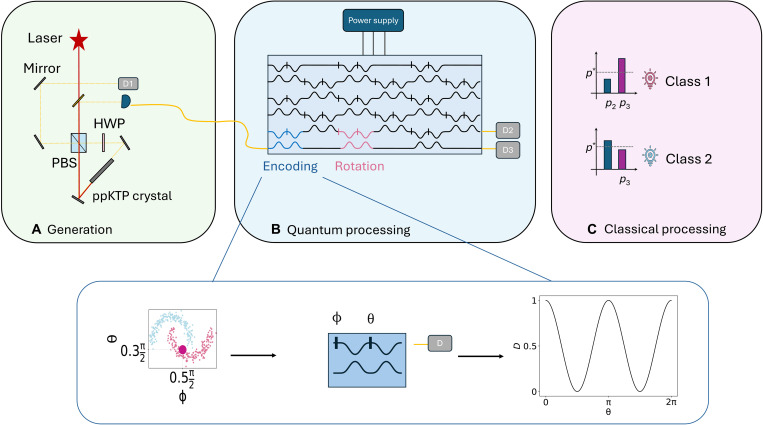
Experimental apparatus. Our algorithm consists of three stages: (**A**) generation of single photons encoding the input qubit state, (**B**) quantum processing, and (**C**) classical postprocessing for classification. (A) Single-photon inputs are produced through a spontaneous parametric down-conversion source based on a periodically poled potassium titanyl phosphate (ppKTP) crystal pumped at 775 nm, together with a half wave plate (HWP) and a polarizing beam splitter (PBS). The source generates separable photon pairs, where one photon is heralding and the other is injected into the photonic circuit. The latter encodes one qubit in the path degree of freedom (dual-rail). (B) The controlled evolution of this qubit is implemented by tuning the phase shifters of the circuit. Each layer of the data reuploading scheme consists of two tunable MZIs: The first encodes the input data, while the second applies a trainable rotation. As illustrated with the moon dataset, a data point with coordinates (*x*_1_ and *x*_2_) is encoded by evolving the input qubit through a MZI whose external and internal phases implement $x_{1}\frac{\pi }{2}$ and $x_{2}\frac{\pi }{2}$, respectively. Because of circuit size limitations, to perform the full experiment, we precomputed the unitary corresponding to long MZI sequences and implemented it through a single effective interferometer. The overall input/output transformation is nonlinear. The output mode in which the photon exits the circuit is detected with single-photon detectors, and we postselect events in which both the trigger and one output detector click simultaneously. (C) The final step is fully classical: The measured output probabilities are processed through a LDA algorithm, which determines a threshold to assign each data point to class 1 or class 2. The training phase can be simulated to find optimal parameters before implementation or performed directly on-chip via a parameter-shift rule. The same output statistics can also be reproduced by injecting coherent light instead of single-photon states.

The experimental implementation of the data reuploading described in the previous sections was achieved with the experimental apparatus shown in Fig. 5. This can be split into three parts: the input generation, the quantum processing, and the classical postprocessing.

The photonic input was generated by a collinear type II spontaneous parametric down conversion source, pumped at 775 nm, emitting degenerate photons pairs at a wavelength of 1550 nm. The nonlinear crystal that was used is a periodically poled titanyl phosphate, placed in a Sagnac interferometer. The crystal was pumped only in one spatial direction to generate the separable state ∣01〉. Then, one photon was only used to herald the generation of the second one that was implementing the qubit state.

For the qubit encoding, we adopt the dual rail scheme, i.e., considering two spatial modes, the presence of one photon (or of the coherent light) on the first mode would implement the state ∣0〉 and, on the second, the state ∣1〉. Therefore, unlike previous implementation that require two-photon states [e.g., (*19*)], our setup operates with single-photon inputs, significantly reducing resource requirements and simplifying the experimental architecture.

Then, the quantum processing was performed through a six-mode universal photonic integrated circuit (*55*), realized via femtosecond laser writing and equipped with thermo-optic phase shifters, for implementing the required unitary transformations (*49*). The input and output ports are directly interfaced with single mode fibers, which are pigtailed to the circuit. Although the used circuit features six input and outputs, we only used a 2 × 2 submatrix of the whole six-mode unitary transformation, corresponding to a single MZI. Let us point out that, although the conceptual scheme of the experiment entails a sequence of encoding and trainable MZIs (whose number depends on the dimensionality of the considered data points and on the number of layers), the actual implementation consists of a single Mach-Zehnder, implementing the overall transformation. Considering the conceptual scheme in Fig. 1, we can evaluate the overall unitary operation of the circuit, in Eq. 2, and implement it on a single interferometer. However, if we consider the state of the art of integrated circuits, the number of input/output modes of fully programmable circuits on borosilicate glass substrates goes up to 24 (*56*). This implies that our smaller-scale classifications would be implementable using a sequence of Mach-Zehnders, reaching high accuracies. Considering different substrates, for silicon nitride–based circuits, the largest reported features 20 modes (*57*) and silicon on silica 26 modes (*58*). However, the last two platforms suffer, respectively, from severe cross-talk (hindering the calibration) and from high coupling losses.

In the end, the two used output modes of the photonic processor are connected to superconducting nanowire single photon detectors. The frequencies of the clicks registered by the two detectors at the output of the chip, i.e., *D*_2_ and *D*_3_, in coincidence with the one detecting the heralding photon (*D*_1_), give the probabilities *p*_2_ and *p*_3_ as $p_{i}=\frac{N_{i,a}}{\sum_{j=1}^{2}N_{j,a}}$.

The output of the circuit, i.e., the variable *p*_2_, for all of the different inputs, is then fed into a LDA, which finds the threshold that best separates the elements belonging to the two classes. Let us point out that, although in our implementation we use single photon inputs, the same output statistics can be obtained, by using a coherent light source.

At the beginning of the procedure, the trainable parameters, i.e., the rotations that are in between the encoding unitaries, are set to random values. Then, during the training phase, these parameters are updated to enhance the accuracy in the reproduction of the training data. This training can be performed via numerical simulations, by using a gradient descent algorithm (*59*) or directly on the quantum platform, where gradients are approximated through a parameter shift rule (*54*) (see note S3). In all numerical simulations, we used the Adam optimizer (*59*) with its default hyperparameter settings as implemented in TensorFlow. For training directly on the photonic hardware, in addition to the adapted gradient acquisition, the learning rate was increased to ensure experimental feasibility.

In the end, after the retrieve of the optimal parameters (i.e., those which minimize the classification error on the training data), we can check the accuracy in the predictions of the model on new unknown data (namely, the test set). This phase is performed by encoding the test data into the circuit and, analogously to before, collecting the output statistics and feeding it into the LDA.

## Supplementary Materials

### This PDF file includes:

Supplementary Text Table S1 Fig. S1 References
