# Supplementary material for: Experimental data reuploading with provable enhanced learning capabilities
Source: Sci Adv. 2026 Apr 10;12(15):eaeb1397. doi: 10.1126/sciadv.aeb1397 (PMC13068056; doi:10.1126/sciadv.aeb1397)
Supplement: Supplementary file 1 — Supplementary Text Table S1 Fig. S1 References [file sciadv.aeb1397_sm.pdf]

Science Advances
AAAS

Supplementary Materials for

Experimental data reuploading with provable enhanced learning capabilities

Martin F. X. Mauser *et al.*

Corresponding author: Martin F. X. Mauser, martin.mauser@univie.ac.at; Iris Agresti, iris.agresti@univie.ac.at; Philip Walther, philip.walther@univie.ac.at

Sci. Adv. **12**, eaeb1397 (2026)
DOI: 10.1126/sciadv.aeb1397

This PDF file includes:

Supplementary Text
Table S1
Fig. S1
References

Supplementary Material

S1. VC DIMENSION OF QUANTUM LEARNING MODELS

In order to determine the VC dimension of the investigated quantum learning models, i.e. the compressed and original data re-uploading scheme, one can try to find a simpler approximation of those systems. In that way, we will find an upper bound. If one is interested in a lower bound of the VC dimension of quantum circuits the reader is referred to [27].

S1.1. Compressed scheme

As mentioned in the main text, each operation on the quantum system will be implemented via a Mach-Zehnder interferometer (MZI), whose action is reported in Eq. (3) of the main text. Hereby, we simplify our notation by setting $\phi_2^E = 0$ and writing $\phi_1^E = \phi$ for the external phase-shifter. Thereby the Jones matrix simplifies to:

$$ie^{i\frac{\theta}{2}}\begin{pmatrix} e^{i\phi}\sin\left(\frac{\theta}{2}\right) & e^{i\phi}\cos\left(\frac{\theta}{2}\right) \\ \cos\left(\frac{\theta}{2}\right) & -\sin\left(\frac{\theta}{2}\right) \end{pmatrix} \tag{1}$$

In order to demonstrate that the compressed scheme does indeed have an infinite VC dimension, we just need to consider the action of the quantum system on one fixed input state and a single layer of MZIs. If we insert the state $|1\rangle$, where $\phi = 0$ and $\theta = \omega \cdot x$ the system evolves as follows:

$$ie^{i\frac{\omega x}{2}}\begin{pmatrix} \sin\left(\frac{\omega x}{2}\right) & \cos\left(\frac{\omega x}{2}\right) \\ \cos\left(\frac{\omega x}{2}\right) & -\sin\left(\frac{\omega x}{2}\right) \end{pmatrix}\begin{pmatrix} 0 \\ 1 \end{pmatrix} = ie^{i\frac{\omega x}{2}}\begin{pmatrix} \cos\left(\frac{\omega x}{2}\right) \\ -\sin\left(\frac{\omega x}{2}\right) \end{pmatrix} \tag{2}$$

Since our measurements are insensitive to global phases, the probability of finding the system in the state $|1\rangle$ after the operation is $p_1 = |-\sin\left(\frac{\omega x}{2}\right)|^2$ and in the state $|0\rangle$ is $p_0 = |\cos\left(\frac{\omega x}{2}\right)|^2$. Typically, one assigns a point x to class 0 if $p_0 > p_1$ and to class 1 otherwise. Furthermore, one can simplify $p_0 = \frac{1}{2}(\cos(\omega x) + 1)$ and $p_1 = \frac{1}{2}(1 - \cos(\omega x))$. For that reason, we can assign the class based on the sign of $p_0 - p_1 = \cos(\omega x)$. Therefore, our set of implementable functions is given by:

$$\mathcal{H} = \left\{ x \mapsto \sin\left(\omega x + \frac{\pi}{2}\right) | \omega \in \mathbb{R} \right\}. \tag{3}$$

Since we know that the VC dimension of the set of sinusoidal functions is infinite [40, Example 3.16], the VC dimension of the compressed scheme is also infinite.

S1.2. Original scheme

In order to estimate the VC dimension of the original scheme, it is more convenient to work with the following (equivalent) Jones matrix of a MZI:

$$\frac{1}{2}\begin{pmatrix} \left(-1 + e^{i\theta}\right)e^{i\phi} & i\left(1 + e^{i\theta}\right) \\ i\left(1 + e^{i\theta}\right)e^{i\phi} & \left(1 - e^{i\theta}\right) \end{pmatrix}. \tag{4}$$

To recap, the action of a single layer in the original scheme $U_L(\vec{x}, \vec{\vartheta})$ is given by two MZIs, one depending only on the data ($\vec{x}$), the other solely on tuneable parameters ($\vec{\vartheta}$), one after the other:

$$\begin{aligned} U_L(\vec{x}, \vec{\vartheta}) &= \frac{1}{4}\begin{pmatrix} \left(-1 + e^{i\vartheta_2}\right)e^{i\vartheta_1} & i\left(1 + e^{i\vartheta_2}\right) \\ i\left(1 + e^{i\vartheta_2}\right)e^{i\vartheta_1} & \left(1 - e^{i\vartheta_2}\right) \end{pmatrix}\begin{pmatrix} \left(-1 + e^{ix_1}\right)e^{ix_2} & i\left(1 + e^{ix_1}\right) \\ i\left(1 + e^{ix_1}\right)e^{ix_2} & \left(1 - e^{ix_1}\right) \end{pmatrix} \\ &= \begin{pmatrix} W_{-+--}(\vec{\vartheta})e^{ix_2} + W_{---+}(\vec{\vartheta})e^{i(x_1+x_2)} & iW_{+-++}(\vec{\vartheta}) + iW_{---+}(\vec{\vartheta})e^{ix_1} \\ iW_{+---}(\vec{\vartheta})e^{ix_2} + iW_{++-+}(\vec{\vartheta})e^{i(x_1+x_2)} & W_{+---}(\vec{\vartheta}) + W_{--+-}(\vec{\vartheta})e^{ix_1} \end{pmatrix} \end{aligned}$$

with:

$$W_{\pm\pm\pm\pm}(\vec{\vartheta}) = \frac{1}{4}\left(\pm 1 \pm e^{i\vartheta_1} \pm e^{i\vartheta_2} \pm e^{i(\vartheta_1+\vartheta_2)}\right)$$

In the simplest example, we evaluate the learning system with one layer and one dimensional data $x_1 = x, \ \ x_2 = 0$. Hereby we will again insert the state $|1\rangle$:

$$\begin{pmatrix} W_{-+--}(\vec{\vartheta}) + W_{---+}(\vec{\vartheta})e^{ix} & iW_{+-++}(\vec{\vartheta}) + iW_{---+}(\vec{\vartheta})e^{ix} \\ iW_{+---}(\vec{\vartheta}) + iW_{++-+}(\vec{\vartheta})e^{ix} & W_{+---}(\vec{\vartheta}) + W_{--+-}(\vec{\vartheta})e^{ix} \end{pmatrix} \begin{pmatrix} 0 \\ 1 \end{pmatrix} = \begin{pmatrix} iW_{+-++}(\vec{\vartheta}) + iW_{---+}(\vec{\vartheta})e^{ix} \\ W_{+---}(\vec{\vartheta}) + W_{--+-}(\vec{\vartheta})e^{ix} \end{pmatrix} \tag{5}$$

Therefore, the probabilities for the measured state after the operation is given by:

$$p_0 = |iW_{+-++}(\vec{\vartheta}) + iW_{---+}(\vec{\vartheta})e^{ix}|^2 \tag{6}$$

$$p_1 = |W_{+---}(\vec{\vartheta}) + W_{--+-}(\vec{\vartheta})e^{ix}|^2. \tag{7}$$

Similar to the derivation in the previous subsection, we are using the sign of $p_0 - p_1$ to assign the class label. However, in addition, we introduce a free parameter t which is given by the threshold found by the Linear Discriminant Analysis (LDA) to maximize the classification accuracy. This yields the following set of implementable functions:

$$\mathcal{H} = \{x \mapsto \text{sign}[a + b\cos x + c\sin x] | a, b, c \in \mathbb{R}\} \tag{8}$$

with

$$a = |W_{+-++}(\vec{\vartheta})|^2 + |W_{---+}(\vec{\vartheta})|^2 - |W_{+---}(\vec{\vartheta})|^2 - |W_{--+-}(\vec{\vartheta})|^2 + t \tag{9}$$

$$b = 2\Re(W_{+-++}(\vec{\vartheta})W^*_{---+}(\vec{\vartheta})) - 2\Re(W_{+---}(\vec{\vartheta})W^*_{--+-}(\vec{\vartheta})) \tag{10}$$

$$c = 2\Im(W^*_{+-++}(\vec{\vartheta})W_{---+}(\vec{\vartheta})) - 2\Im(W^*_{+---}(\vec{\vartheta})W_{--+-}(\vec{\vartheta})). \tag{11}$$

Since we are mainly interested in an upper bound for the VC dimension, we can assume that $a, b,$ and c are free tunable parameters. Furthermore these parameters can be simplified to the following:

$$a = t \tag{12}$$

$$b = -\cos(\vartheta_2) \tag{13}$$

$$c = \frac{1}{2}(\sin(\vartheta_1 + \vartheta_2) - \sin(\vartheta_1 - \vartheta_2)) \tag{14}$$

Further, one can rewrite the evaluation of the VC dimension

$$a + b\cos x + c\sin x = a + R\cos(x - \varphi)$$

with

$$R = \sqrt{b^2 + c^2} \text{ and } \varphi = \arctan\left(\frac{c}{b}\right).$$

Assuming that $|a| \leq R$, we can identify the 2π periodic transition points of the sign of the function:

$$\alpha = \varphi + \arccos\left(-\frac{a}{R}\right) \quad \text{or} \quad \beta = \varphi + 2\pi - \arccos\left(-\frac{a}{R}\right)$$

Therefore, the sign of the function is positive for $x \in (\alpha, \beta) \mod 2\pi$ and negative for $x \in (\beta, \alpha + 2\pi) \mod 2\pi$. One can now write the set of implementable functions as follows:

$$\mathcal{H} = \{x \mapsto \mathbb{1}_{\mathcal{S}=[\alpha+2\pi n, \beta+2\pi n]} | \alpha, \beta \in \mathbb{R}; \forall n \in \mathbb{Z}\}. \tag{15}$$

This implies that all the points in the interval $[a + 2\pi n, b + 2\pi n]$ are classified as belonging to class 1 and those outside to class 2. In contrast if $|a| > R$, a constant function is implemented. It is easy to see that for such a system the VC dimension is 3, since we already know that the VC dimension of a single one-dimensional interval is 2 [40, Example 3.11] and the periodicity of the system extents the VC dimension by one. A detailed proof of this (as well as the extension to more layers) can be found in the main text.

The same analysis can be extended to multiple layers, either still with one-dimensional inputs x or generalized to higher dimensional input data. For simplicity, we will limit the data encoding to be one-dimensional for each encoding MZI, but note that behavior of the learning system is similar when using both phases of the MZI to encode data. Note that this statement is true for every layer except for the very first, where the apparatus is intrinsically insensitive to the external phase of the MZI, and thereby to one of the two encoded phases - the one denoted x_2 in $U_L(\vec{x}, \vec{\vartheta})$.

The quantum learning system with two layers can therefore be written as:

$$U_L(x_2,\vec{\vartheta_2})U_L(x_1,\vec{\vartheta_1}) = \begin{pmatrix} W_{-+--}(\vec{\vartheta_2}) + W_{---+}(\vec{\vartheta_2})e^{ix_2} & iW_{+-++}(\vec{\vartheta_2}) + iW_{---+}(\vec{\vartheta_2})e^{ix_2} \\ iW_{+---}(\vec{\vartheta_2}) + iW_{++-+}(\vec{\vartheta_2})e^{ix_2} & W_{+---}(\vec{\vartheta_2}) + W_{--+-}(\vec{\vartheta_2})e^{ix_2} \end{pmatrix} \cdot \begin{pmatrix} W_{-+--}(\vec{\vartheta_1}) + W_{---+}(\vec{\vartheta_1})e^{ix_1} & iW_{+-++}(\vec{\vartheta_1}) + iW_{---+}(\vec{\vartheta_1})e^{ix_1} \\ iW_{+---}(\vec{\vartheta_1}) + iW_{++-+}(\vec{\vartheta_1})e^{ix_1} & W_{+---}(\vec{\vartheta_1}) + W_{--+-}(\vec{\vartheta_1})e^{ix_1} \end{pmatrix}$$

Therefore, the probabilities for the measured state after the operation is given by:

$$\begin{aligned} p_0 = |&(W_{-+--}(\vec{\vartheta_2}) + W_{---+}(\vec{\vartheta_2})e^{ix_2})(iW_{+-++}(\vec{\vartheta_1}) + iW_{---+}(\vec{\vartheta_1})e^{ix_1}) + \\ &(iW_{+-++}(\vec{\vartheta_2}) + iW_{---+}(\vec{\vartheta_2})e^{ix_2})(W_{+---}(\vec{\vartheta_1}) + W_{--+-}(\vec{\vartheta_1})e^{ix_1})|^2 \\ p_1 = |&(iW_{+---}(\vec{\vartheta_2}) + iW_{++-+}(\vec{\vartheta_2})e^{ix_2})(iW_{+-++}(\vec{\vartheta_1}) + iW_{---+}(\vec{\vartheta_1})e^{ix_1}) + \\ &(W_{+---}(\vec{\vartheta_2}) + W_{--+-}(\vec{\vartheta_2})e^{ix_2})(W_{+---}(\vec{\vartheta_1}) + W_{--+-}(\vec{\vartheta_1})e^{ix_1})|^2 \end{aligned}$$

If both layers share the same data input, i.e. $x_1 = x_2 = x$, then the system can approximated by the following set of implementable functions:

$$\mathcal{H} = \{x \mapsto a + b\cos(x) + c\sin(x) + d\cos(2x) + e\sin(2x) | a,b,c,d,e \in \mathbb{R}\} \tag{16}$$

with additional constraints on the parameters (a, b, c, d and, e), which areimplied by the particular implementation of the system. The upper bound of the VC dimension of the learning system, as mentioned in the main text, therefore increases from dimension 3 to dimension 5. Thereby, one can see that the upper bond of the VC dimension, as also hypothesized by the experimental results, grows with increasing amount of layers.

If, however, the two layers do not share the same input data, in order to implement higher-dimensional data, the system can be approximately described by the following set of implementable functions with constraint parameters:

$$\mathcal{H} = \{(x_1,x_2) \mapsto a + b\cos(x_1) + c\sin(x_1) + d\cos(x_2) + e\sin(x_2) + f\cos(x_1+x_2) + g\sin(x_1+x_2) |\ a,b,c,d,e,f,g \in \mathbb{R}\}. \tag{17}$$

The generalization to higher-dimensional input data thereby shows that the output function depends non-linearly on each of the individual input dimensions and on their sum.

Both of these systems can again be written in the form of a periodic interval classifier in a similar manner to the simple one-dimensional case.

S2. UNIVERSAL APPROXIMATION THEOREM

The universal approximator property of the original model was recently shown in [31], analogously to the compressed scheme [10, 15]. First, we demonstrate this by analyzing the approximated set of implementable functions. Any function that is periodic, e.g. 2π-periodic, and continuous can be represented in terms of the Fourier series, as follows:

$$g(x) = \frac{a_0}{2} + \sum_{k=1}^{\infty} [a_k \cos(kx) + b_k \sin(kx)]. \tag{18}$$

Furthermore, we also know from the best approximation properties of the Fourier series, see for example [60, Lemma 1.2], that the truncated Fourier series yields the best possible approximation of any periodic square integrable function. Trigonometric polynomials are, therefore, dense in the space of continuous periodic functions with respect to the uniform norm. Similarly, the general expression of the N-layered learning model in the derived approximation picture is:

$$\mathcal{H} = \left\{ x \mapsto a + \sum_{l=1}^{N} [b_l \cos(lx) + c_l \sin(lx)] \;\middle|\; a, b_l, c_l \in \mathbb{R} \right\} \tag{19}$$

By allowing $N \to \infty$, the learning model is equivalent to the Fourier series representation of any continuous periodic function. Thereby, also the original implementation of the learning model is a universal approximation for continuous functions on a compact interval.

S3. ON-CHIP AND NUMERICAL TRAINING PROCEDURE

The models shown in this publications were all trained in a similar fashion. Hereby, we mainly utilised the python libraries TensorFlow [61] and Strawberry Fields [62]. We implemented a train of universal Mach-Zehnder Interferometers (MZIs) to encode the data and the tunable parameters and simulated the propagation with single photons, or equivalently coherent light, through the system. In the training process, we used the Adam optimizer as implemented by TensorFlow with default parameters. As a loss function a linear discriminant analysis (LDA) loss [63] was used and a LDA was used for assigning the class labels. The entire system was then trained to convergence, for a maximum of 10.000 iterations. The necessary gradients were calculated via automatic differentiation.

Furthermore, we also investigated the training on the experimental hardware. This was implemented via the parameter shift rule [54]. In this context, we also investigated the performance of a ”forward”, ”backward”, and ”central” implementation. We found out that, for the investigated datasets, a combination of ”backward” finite differences and ”central” finite difference, yielded the best ratio of experimental runtime (number of runs per gradient estimation) and training performance. While the architecture was kept unchanged, except for the derivation of the gradients, the learning rate was adapted the yield faster convergence in order to ensure experimental feasibility.

S4. CONTRIBUTION OF LDA TO CLASSIFICATION PERFORMANCE

The role of Linear Discriminant Analysis (LDA) in our pipeline is limited to providing an adjustable threshold for classification. Without LDA, classifying would rely on a simple majority rule, i.e. if the probability of detecting a photon in mode 1 exceeds that in mode 2, the corresponding point is classified as class 1; otherwise to class 2. The LDA introduces a tunable threshold t, so a datapoint is assigned to class 1 only if the probability of detecting photons in mode 1 exceeds t. Since the LDA operates in a one-dimensional space, it is computationally inexpensive and does not account for the non-linear separability of the data.

Figure S1 illustrate the impact of the LDA on classification accuracy for the circles dataset. Two quantum data re-uploading models were trained with identical parameters, one using LDA and one without (threshold fixed at 0.5). For the latter, the loss function was adjusted from the LDA loss [63] to a distance-based measure. Removing the LDA eliminates the constant offset introduced by the adjustable, as also reflected in eq. 9 in the VC dimension analysis, and slightly reduces accuracy. However, the resulting feature map continues to provide a substantial improvement over the classical baseline, and this advantage grows with the number of layers even without LDA. This demonstrates that the observed performance gains are primarily due to the non-linear processing enabled by the quantum data re-uploading protocol rather than the contribution of the LDA.

To confirm that the addition of non-linear features is beneficial for the task, we benchmarked several classical classifiers on the same dataset (see table S1). Linear models such as LDA, logistic regression, and linear Support Vector Machine (SVM) fail to achieve high accuracy, while non-linear classifiers (e.g., polynomial and RBF SVM, random forest, k-Nearest Neighbors) perform well. This confirms that the high accuracy achieved by our approach is enabled by the non-linear processing introduced by the quantum data re-uploading model.

linear classifier	accuracy (training)	accuracy (testing)
Linear Discriminant Analysis	58.75%	73.00%
Logistic Regression	56.25%	70.00%
Linear SVM	52.25%	41.00%
non-linear classifier	accuracy (training)	accuracy (testing)
Polynolial SVM (degree = 3)	72.25%	70.00%
Polynolial SVM (degree = 4)	86.25%	91.00%
Random Forrest	100.00%	97.00%
k-Nearest Neighbors (k = 5)	100.00%	100.00%
RBF SVM	100.00%	100.00%

TABLE S1: Comparison of classical linear and non-linear classifiers on the circles dataset. Linear models such as LDA, logistic regression, and a linear Support Vector Machine (SVM) fail to achieve high accuracy, while non-linear classifiers (e.g., polynomial and RBF SVM, random forest, k-Nearest Neighbors) perform well. This highlights that the high accuracy achieved by the quantum data re-uploading model cannot be attributed to LDA alone, but rather to the non-linear processing introduced by the quantum feature map.

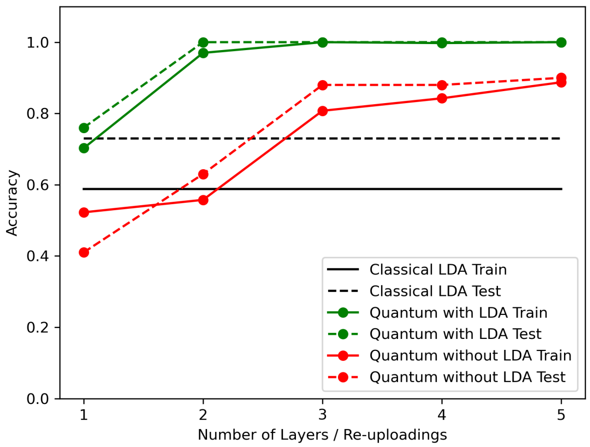

FIG. S1: Comparison of the contribution of Linear Discriminant Analysis (LDA) to the overall classification performance on the circles dataset. Two quantum data re-uploading models were trained with identical parameters, but with a different loss function, since directly used the separability with a LDA as the loss function: one using LDA (green curves) and one without (red curves). Removing the LDA corresponds to fixing the classification threshold at 0.5, which eliminates the constant offset provided by LDA and reduces accuracy. Both training and testing accuracies are shown as a function of the number of layers (re-uploadings), demonstrating that the quantum feature map significantly improves even without LDA.

REFERENCES

1. P. W. Shor, “Algorithms for quantum computation: Discrete logarithms and factoring,” in *Proceedings 35th Annual Symposium on Foundations of Computer Science* (IEEE, 1994), pp. 124–134.

2. L. K. Grover, “A fast quantum mechanical algorithm for database search,” in *Proceedings of the Twenty-eighth Annual ACM Symposium on Theory of Computing* (Association for Computing Machinery; 1996), pp. 212–219.

3. S. Aaronson, A. Arkhipov, “The computational complexity of linear optics,” in *Proceedings of the Forty-third Annual ACM Symposium on Theory of Computing* (Association for Computing Machinery; 2011), pp. 333–342.

4. A. P. Lund, M. J. Bremner, T. C. Ralph, Quantum sampling problems, BosonSampling and quantum supremacy. *npj Quantum Inf.* **3**, 15 (2017).

5. F. Arute, K. Arya, R. Babbush, D. Bacon, J. C. Bardin, R. Barends, R. Biswas, S. Boixo, F. G. S. L. Brandao, D. A. Buell, B. Burkett, Y. Chen, Z. Chen, B. Chiaro, R. Collins, W. Courtney, A. Dunsworth, E. Farhi, B. Foxen, A. Fowler, C. Gidney, M. Giustina, R. Graff, K. Guerin, S. Habegger, M. P. Harrigan, M. J. Hartmann, A. Ho, M. Hoffmann, T. Huang, T. S. Humble, S. V. Isakov, E. Jeffrey, Z. Jiang, D. Kafri, K. Kechedzhi, J. Kelly, P. V. Klimov, S. Knysh, A. Korotkov, F. Kostritsa, D. Landhuis, M. Lindmark, E. Lucero, D. Lyakh, S. Mandrà, J. R. McClean, M. McEwen, A. Megrant, X. Mi, K. Michielsen, M. Mohseni, J. Mutus, O. Naaman, M. Neeley, C. Neill, M. Y. Niu, E. Ostby, A. Petukhov, J. C. Platt, C. Quintana, E. G. Rieffel, P. Roushan, N. C. Rubin, D. Sank, K. J. Satzinger, V. Smelyanskiy, K. J. Sung, M. D. Trevithick, A. Vainsencher, B. Villalonga, T. White, Z. J. Yao, P. Yeh, A. Zalcman, H. Neven, J. M. Martinis, Quantum supremacy using a programmable superconducting processor. *Nature* **574**, 505–510 (2019).

6. H.-S. Zhong, H. Wang, Y.-H. Deng, M.-C. Chen, L.-C. Peng, Y.-H. Luo, J. Qin, D. Wu, X. Ding, Y. Hu, P. Hu, X.-Y. Yang, W.-J. Zhang, H. Li, Y. Li, X. Jiang, L. Gan, G. Yang, L. You, Z. Wang, L. Li, N. L. Liu, C.-Y. Lu, J.-W. Pan, Quantum computational advantage using photons. *Science* **370**, 1460–1463 (2020).

7. L. S. Madsen, F. Laudenbach, M. F. Askarani, F. Rortais, T. Vincent, J. F. F. Bulmer, F. M. Miatto, L. Neuhaus, L. G. Helt, M. J. Collins, A. E. Lita, T. Gerrits, S. W. Nam, V. D. Vaidya, M. Menotti, I. Dhand, Z. Vernon, N. Quesada, J. Lavoie, Quantum computational advantage with a programmable photonic processor. *Nature* **606**, 75–81 (2022).

8. J. Biamonte, P. Wittek, N. Pancotti, P. Rebentrost, N. Wiebe, S. Lloyd, Quantum machine learning. *Nature* **549**, 195–202 (2017).

9. V. Dunjko, H. J. Briegel, Machine learning & artificial intelligence in the quantum domain: A review of recent progress. *Rep. Prog. Phys.* **81**, 074001 (2018).

10. A. Pérez-Salinas, A. Cervera-Lierta, E. Gil-Fuster, J. I. Latorre, Data re-uploading for a universal quantum classifier. *Quantum* **4**, 226 (2020).

11. M. Schuld, F. Petruccione, M. Schuld, F. Petruccione, "Quantum models as kernel methods," in *Machine Learning with Quantum Computers* (Springer, Cham; 2021), pp. 217–245.

12. Y. Liu, S. Arunachalam, K. Temme, A rigorous and robust quantum speed-up in supervised machine learning. *Nat. Phys.* **17**, 1013–1017 (2021).

13. H.-Y. Huang, M. Broughton, M. Mohseni, R. Babbush, S. Boixo, H. Neven, J. R. McClean, Power of data in quantum machine learning. *Nat. Commun.* **12**, 2631 (2021).

14. S. Jerbi, L. K. Fiderer, H. P. Nautrup, J. M. Kübler, H. J. Briegel, V. Dunjko, Quantum machine learning beyond kernel methods. *Nat. Commun.* **14**, 514 (2023).

15. T. Goto, Q. H. Tran, K. Nakajima, Universal approximation property of quantum machine learning models in quantum-enhanced feature spaces. *Phys. Rev. Lett.* **127**, 090506 (2021).

16. W. K. Wootters, W. H. Zurek, A single quantum cannot be cloned. *Nature* **299**, 802–803 (1982).

17. M. Schuld, R. Sweke, J. J. Meyer, Effect of data encoding on the expressive power of variational quantum-machine-learning models. *Phys. Rev. A* **103**, 032430 (2021).

18. Z. Yu, H. Yao, M. Li, X. Wang, Power and limitations of single-qubit native quantum neural networks. *Adv. Neural Inf. Proces. Syst.* **35**, 27810–27823 (2022).

19. T. Ono, W. Roga, K. Wakui, M. Fujiwara, S. Miki, H. Terai, M. Takeoka, Demonstration of a bosonic quantum classifier with data reuploading. *Phys. Rev. Lett.* **131**, 013601 (2023).

20. A. Pérez-Salinas, D. López-Núñez, A. García-Sáez, P. Forn-Díaz, J. I. Latorre, One qubit as a universal approximant. *Phys. Rev. A* **104**, 012405 (2021).

21. L. Fan, H. Situ, Compact data encoding for data re-uploading quantum classifier. *Quantum Inf. Process.* **21**, 87 (2022).

22. N. L. Wach, M. S. Rudolph, F. Jendrzejewski, S. Schmitt, Data re-uploading with a single qudit. *Quantum Mach. Intell.* **5**, 36 (2023).

23. P. Easom-Mccaldin, A. Bouridane, A. Belatreche, R. Jiang, On depth, robustness and performance using the data re-uploading single-qubit classifier. *IEEE Access* **9**, 65127–65139 (2021).

24. T. Dutta, A. Pérez-Salinas, J. P. S. Cheng, J. I. Latorre, M. Mukherjee, Single-qubit universal classifier implemented on an ion-trap quantum device. *Phys. Rev. A* **106**, 012411 (2022).

25. T. Dutta, A. Jin, C. L. Huihong, J. I. Latorre, M. Mukherjee, Practicality of training a quantum-classical machine in the noisy intermediate-scale quantum era. *iScience* **28**, 113058 (2025).

26. M. C. Caro, E. Gil-Fuster, J. J. Meyer, J. Eisert, R. Sweke, Encoding-dependent generalization bounds for parametrized quantum circuits. *Quantum* **5**, 582 (2021).

27. C.-C. Chen, M. Sogabe, K. Shiba, K. Sakamoto, T. Sogabe, General Vapnik–Chervonenkis dimension bounds for quantum circuit learning. *J. Phys. Complex.* **3**, 045007 (2022).

28. E. Gil-Fuster, J. Eisert, C. Bravo-Prieto, Understanding quantum machine learning also requires rethinking generalization. *Nat. Commun.* **15**, 2277 (2024).

29. C. Pentangelo, N. di Giano, S. Piacentini, R. Arpe, F. Ceccarelli, A. Crespi, R. Osellame, High-fidelity and polarization-insensitive universal photonic processors fabricated by femtosecond laser writing. *Nanophotonics* **13**, 2259–2270 (2024).

30. G. Corrielli, A. Crespi, R. Osellame, Femtosecond laser micromachining for integrated quantum photonics. *Nanophotonics* **10**, 3789–3812 (2021).

31. A. Pérez-Salinas, M. Y. Rad, A. Barthe, V. Dunjko, Universal approximation of continuous functions with minimal quantum circuits. arXiv:2411.19152 [quant-ph] (2024).

32. R. Hamerly, L. Bernstein, A. Sludds, M. Soljačić, D. Englund, Large-scale optical neural networks based on photoelectric multiplication. *Phys. Rev. X* **9**, 021032 (2019).

33. T. Hastie, R. Tibshirani, J. Friedman, J. Franklin, "The elements of statistical learning: Data mining, inference and prediction," in *The Mathematical Intelligencer* (Springer Science+Business Media; 2005), vol. 27, pp. 83–85.

34. M. Periyasamy, N. Meyer, C. Ufrecht, D. D. Scherer, A. Plinge, C. Mutschler, "Incremental data-uploading for full-quantum classification," in *2022 IEEE International Conference on Quantum Computing and Engineering (QCE)* (IEEE, 2022), pp. 31–37.

35. V. N. Vapnik, A. Y. Chervonenkis, "On the uniform convergence of relative frequencies of events to their probabilities," in *Measures of complexity: Festschrift for alexey chervonenkis* (Springer, 2015), pp. 11–30.

36. P. Petersen, J. Zech, Mathematical theory of deep learning. arXiv:2407.18384 [cs.LG] (2024).

37. S. Shalev-Shwartz, S. Ben-David, *Understanding Machine Learning: From Theory to Algorithms* (Cambridge Univ. Press, 2014); 10.1017/CBO9781107298019.

38. L. G. Valiant, A theory of the learnable. *Commun. ACM* **27**, 1134–1142 (1984).

39. H. Li, Z. Xu, G. Taylor, C. Studer, T. Goldstein, Visualizing the loss landscape of neural nets. arXiv.1712.09913 [cs.LG] (2018).

40. M. Mohri, A. Rostamizadeh, A. Talwalkar, *Foundations of Machine Learning (Second Edition)* (MIT Press; 2018).

41. S. Hochreiter, J. Schmidhuber, Flat Minima. *Neural Comput.* **9**, 1–42 (1997).

42. N. Keskar, D. Mudigere, J. Nocedal, M. Smelyanskiy, P. T. P. Tang, On large-batch training for deep learning: Generalization gap and sharp minima. arXiv:1609.04836 [cs.LG] (2016).

43. P. Chaudhari, A. Choromanska, S. Soatto, Y. LeCun, C. Baldassi, C. Borgs, J. Chayes, L. Sagun, R. Zecchina, Entropy-SGD: Biasing gradient descent into wide valleys. *J. Stat. Mech.* **2019**, 124018 (2019).

44. P. Marion, L. Chizat, Deep linear networks for regression are implicitly regularized towards flat minima. arXiv:2405.13456 [stat.ML] (2024).

45. F. Pedregosa, G. Varoquaux, A. Gramfort, V. Michel, B. Thirion, O. Grisel, M. Blondel, P. Prettenhofer, R. Weiss, V. Dubourg, J. Vanderplas, A. Passos, D. Cournapeau, M. Brucher, M. Perrot, É. Duchesnay, Scikit-learn: Machine learning in Python. *J. Mach. Learn. Res.* **12**, 2825–2830 (2011).

46. D. Noever, S. E. M. Noever, Overhead MNIST: A benchmark satellite dataset. arXiv:2102.04266 [cs.CV] (2021).

47. I. T. Jolliffe, *Principal Component Analysis*, Springer Series in Statistics (Springer, 1986); 10.1007/978-1-4757-1904-8.

48. K. Pearson, LIII. On lines and planes of closest fit to systems of points in space. *Lond. Edinb. Dubl. Philos. Mag. J. Sci.* **2**, 559–572 (1901).

49. F. Ceccarelli, S. Atzeni, C. Pentangelo, F. Pellegatta, A. Crespi, R. Osellame, Low power reconfigurability and reduced crosstalk in integrated photonic circuits fabricated by femtosecond laser micromachining. *Laser Photonics Rev.* **14**, 2000024 (2020).

50. P. L. McMahon, Nonlinear computation with linear systems. *Nat. Phys.* **20**, 1365–1366 (2024).

51. P. Rodriguez-Grasa, Y. Ban, M. Sanz, Training embedding quantum kernels with data re-uploading quantum neural networks. arXiv:2401.04642 [quant-ph] (2024).

52. D. Freinberger, J. Lemmel, R. Grosu, S. Jerbi, A quantum-classical reinforcement learning model to play atari games. arXiv:2412.08725 [quant-ph] (2024).

53. G. Facelli, D. D. Roberts, H. Wallner, A. Makarovskiy, Z. Holmes, W. R. Clements, Exact gradients for linear optics with single photons. arXiv:2409.16369 [quant-ph] (2024).

54. M. Schuld, V. Bergholm, C. Gogolin, J. Izaac, N. Killoran, Evaluating analytic gradients on quantum hardware. *Phys. Rev. A* **99**, 032331 (2019).

55. W. R. Clements, P. C. Humphreys, B. J. Metcalf, W. S. Kolthammer, I. A. Walmsley, Optimal design for universal multiport interferometers. *Optica* **3**, 1460–1465 (2016).

56. A. Barzaghi, M. Bénéfice, F. Ceccarelli, G. Corrielli, V. Galli, M. Gardina, V. Grimaldi, J. Kaczorowski, F. Malaspina, R. Osellame, C. Pentangelo, A. Rocchetto, A. Rudi, A low-loss, 24-mode laser-written universal photonic processor in a glass-based platform. arXiv:2505.01609 [quant-ph] (2025).

57. C. Taballione, M. C. Anguita, M. Goede, P. Venderbosch, B. Kassenberg, H. Snijders, N. Kannan, W. L. Vleeshouwers, D. Smith, J. P. Epping, R. van der Meer, P. W. H. Pinkse, H. van den Vlekkert, J. J. Renema, 20-mode universal quantum photonic processor. *Quantum* **7**, 1071 (2023).

58. V. Saggio, B. E. Asenbeck, A. Hamann, T. Strömberg, P. Schiansky, V. Dunjko, N. Friis, N. C. Harris, M. Hochberg, D. Englund, S. Wölk, H. J. Briegel, P. Walther, Experimental quantum speed-up in reinforcement learning agents. *Nature* **591**, 229–233 (2021).

59. D. P. Kingma, J. Ba, Adam: A method for stochastic optimization. arXiv:1412.6980 [cs.LG] (2017).

60. E. M. Stein, R. Shakarchi, E. M. Stein, *Fourier Analysis: An Introduction, no. 1 in Princeton Lectures in Analysis*, E. M. Stein, R. Shakarchi, Eds. (Princeton Univ. Press, 15. druck ed., 2003).

61. M. Abadi, A. Agarwal, P. Barham, E. Brevdo, Z. Chen, C. Citro, G. S. Corrado, A. Davis, J. Dean, M. Devin, S. Ghemawat, I. Goodfellow, A. Harp, G. Irving, M. Isard, Y. Jia, R. Jozefowicz, L. Kaiser, M. Kudlur, J. Levenberg, D. Mané, R. Monga, S. Moore, D. Murray, C. Olah, M. Schuster, J. Shlens, B. Steiner, I. Sutskever, K. Talwar, P. Tucker, V. Vanhoucke, V. Vasudevan, F. Viégas, O. Vinyals, P. Warden, M. Wattenberg, M. Wicke, Y. Yu, X. Zheng, TensorFlow: Large-Scale Machine Learning on Heterogeneous Systems (2015); https://tensorflow.org/.

62. N. Killoran, J. Izaac, N. Quesada, V. Bergholm, M. Amy, C. Weedbrook, Strawberry fields: A software platform for photonic quantum computing. *Quantum* **3**, 129 (2019).

63. M. Dorfer, R. Kelz, G. Widmer, Deep linear discriminant analysis. arXiv:1511.04707 [cs.LG] (2016).
